# Avellis syndrome with ipsilateral prosopalgia, glossopharyngeal neuralgia, and central post-stroke pain: A case report and literature review

**DOI:** 10.1097/MD.0000000000030669

**Published:** 2022-09-30

**Authors:** Sijin He, Qigang Chen, Zhicong Jing, Lihua Gu, Kaixuan Luo

**Affiliations:** a Rehabilitation Department, Kunming Municipal Hospital of Traditional Chinese Medicine, Kunming, China; b Department of Physical Therapy, the School of Rehabilitation, Kunming, China.

**Keywords:** Avellis syndrome, central post-stroke pain, glossopharyngeal neuralgia, prosopalgia, stroke

## Abstract

Avellis syndrome is a rare bulbar syndrome. The main lesions may involve nucleus ambiguus and the lateral spinothalamic tract. The typical reported clinical manifestations are hoarseness, dysphagia, pain, and temperature disturbance of contralateral body. The manifestations, however, may vary. We aim to report new manifestations of Avellis syndrome in this report.

**Patient concerns:**

A 47-year-old Chinese peasant woman who felt sudden dizziness, nausea when she was doing the laundry was referred to our department from other hospital. She vomited the stomach contents once and complained numbness of the left trunk and limbs as well as coughing while drinking. The patient presented with palatopharyngeal paralysis, Horner syndrome, and diminished pain as well as temperature sensation in the contralateral face, trunk, and limbs. She also presented with ipsilateral prosopalgia, glossopharyngeal neuralgia, and central poststroke pain.

**Diagnoses:**

T2-weighted MRI images demonstrated a high-signal intensity lesion in the right medulla oblongata which indicated a banded infarction site. The patient was diagnosed with medulla oblongata infarction, Avellis syndrome, Horner syndrome, dysphagia, hemiparesthesia, ipsilateral prosopalgia, glossopharyngeal neuralgia, and central poststroke pain.

**Interventions:**

The patient was administrated aspirin to prevent the aggregation of platelet and rosuvastatin tablets to regulate lipids as well as to stabilize vascular plaque. She was injected with butylphthalide sodium chloride to improve nerve nutritional status and carbamazepine was prescribed to deal with prosopalgia and glossopharyngeal neuralgia. Gabapentin and pregabalin were administrated to deal with the central poststroke pain.

**Outcomes:**

The symptoms of prosopalgia as well as glossopharyngeal neuralgia were gone, and dizziness, dysphagia, and Horner syndrome were significantly alleviated when she was discharged from the hospital while the interventions showed little effect on central poststroke pain.

**Lessons:**

We reported a case of Avellis syndrome who manifested as the typical reported manifestations. The patient, what’s more, presented with ipsilateral trigeminal, glossopharyngeal neuralgia, and central poststroke pain which were described for the first time. It is of great significance for clinicians to recognize the typical as well as other manifestations which helps to make a clear diagnosis.

## 1. Introduction

Avellis syndrome is a rare clinical syndrome which was first reported by German laryngologist Avellis in 1891 and is also known as spinothalamic fascicular-hypothalamic syndrome.^[1]^ The etiology of Avellis syndrome varies, with oblongata infarction that resulted from atherosclerosis being the most common cause.^[2]^ Other etiological factors include Borrelia burgdorferi infection,^[3]^ craniocerebral injury,^[4]^ neurobrucellosis small vessel vasculitis^[5]^, and ulcerative colitis.^[6]^ The most frequently reported clinical manifestations of Avellis syndrome are hoarseness, dysphagia, pain and temperature disturbance of the contralateral body which may due to the damage to the nucleus ambiguous, lateral spinothalamic tract and surrounding nervous nuclei and tracts.^[2–6]^ Other manifestations, however, may be presented in Avellis syndrome according to the varied lesion sites. It is of great importance to distinguish the uncommon clinical manifestations from the common ones in terms of accurate diagnosis of Avellis syndrome. We reported a 47-year-old woman with Avellis syndrome after stroke who presented common reported manifestations as well as the uncommon ones, that is, trigeminal neuralgia (TN), glossopharyngeal neuralgia (GPN), and central pain after stroke. Written informed consent was obtained from the patients for publication of this case report and any accompanying images following the principles outlined in the Helsinki Declaration.

## 2. Case presentation

A 47-year-old Chinese peasant woman who received junior high school education felt sudden dizziness, nausea when she was doing the laundry on December 14, 2020 and was admitted into the local county hospital. The patient vomited the stomach contents once and complained numbness of the left trunk and limbs as well as coughing while drinking. Symptoms such as blurred consciousness, limb weakness, vision rotation, double vision, and limb convulsions were not observed. Computer tomography scan of the brain was carried out after admission and the possible diagnosis of cerebral infarction was proposed. The patient was transferred to the local “state hospital of traditional Chinese medicine” on December 17, 2020, and the brain magnetic resonance imaging (MRI) revealed “infarction of the right medulla oblongata (MO).” Symptomatic treatments were carried to nourish nerve and promote blood circulation. The symptoms, however, were not improved. What’s more, she felt sudden electric shock-like pain in the right face, ear root and throat which lasted for about 20 minutes and relieved by itself gradually on December 20, 2020. No tinnitus, deafness, hearing loss, and other symptoms occurred during the attack.

The patient was referred to our hospital on December 21, 2020 for further diagnosis and treatment. The patient denied the history of headache, family genetic diseases, smoking, and drinking. The history of hypertension, diabetes, coronary heart disease, rheumatic immune diseases, and other systemic diseases was also repudiated.

### 2.1. Physical examination

Body temperature: 36.7°C, pulse: 80 beats/min, respiratory rate: 18 beats/min, blood pressure: 123/88 mm Hg. Obvious abnormality of the heart, lungs, and abdomen were not observed.

### 2.2. Neurological examination

The patient’s examination revealed consciousness and she spoke fluently. The diameters of left and right pupil were 3.5 and 2.5 mm respectively. The light reflex was sensitive. There were obvious blepharoptosis and enophthalmos in her right side. Both eyes moved flexibly in all directions without nystagmus. Her nasolabial grooves were normal and symmetrical. She was able to form bilateral frontal wrinkles, and turn the head laterally to both sides as well as shrug the shoulder vigorously and symmetrically. Her tongue was centered and obvious tongue muscle atrophy as well as tremor were absent. The pharyngeal reflex of the right side was weakened and there was a limited lift of the right soft palate. The uvula was displaced to the left side and the water swallow test was of grade 2. Decreased pain and warm sensation were found in her left face, limbs, and trunk while the bilateral deep sensations were examined normal. The muscle strength score of the four limbs was 5 (manual muscle test) with normal muscle tone. The tendon reflexes existed symmetrically with no pathologic reflexes found to be positive bilaterally. The coordinate movements were normal and meningeal irritation signs were absent.

Auxiliary examination after admission: triglyceride: 2.13 mmol/L; the blood and urine routine, stool routine + occult blood, the function of liver and kidney, the indicators for fibrinolysis, and examination results of cardiac biomarkers were within normative range. The results of carotid duplex ultrasound, transcranial doppler sonography, cardiac color ultrasound, color doppler angiography of extremities, and electrocardiography were normal.

**Figure 1.**
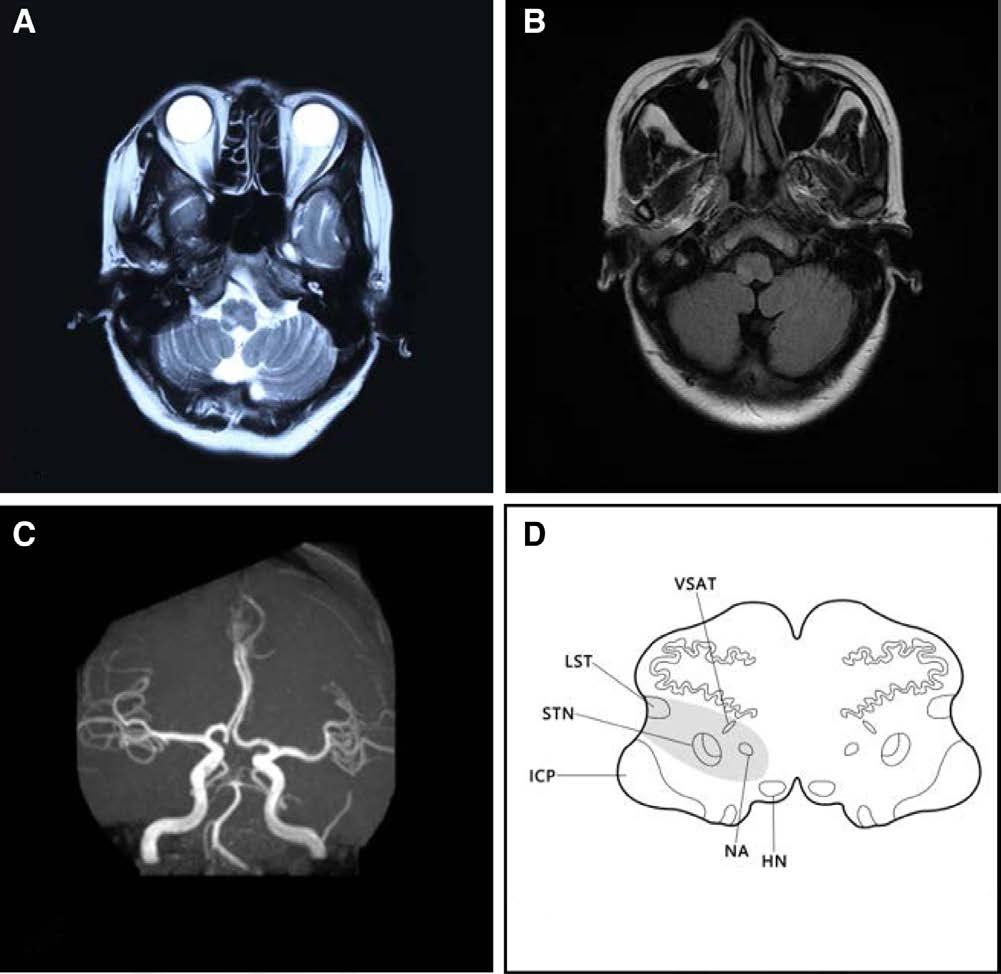
T2-weighted MRI images on December 17, 2020 demonstrated a high-signal lesion in the right medulla oblongata which indicating a banded infarction site (A); FLAIR images of brain MRI on T2 sequences on December 22, 2020 showed slight high-signal in the infarcted site (B); MRA on December 22, 2020 demonstrated that the left dominant vertebral artery which tortuously to the right with no abnormality was observed in the remaining cerebrovascular vessels (C); the simulated diagram of the patient’s lesioned area shows the affected NA, LST, VSAT, and STN. FLAIR = fluid-attenuated inversion recovery, HN = hypoglossal nucleus, ICP = inferior cerebellar peduncle, LST = lateral spinothalamic tract, MRA = magnetic resonance angiography, MRI = magnetic resonance imaging, NA = nucleus ambiguous, STN = spinal trigeminal nucleus, VSAT = ventral trigeminothalamic tract (ventral secondary ascending tract of trigeminal nucleus).

T2-weighted MRI images on December 17, 2020 demonstrated a high-signal intensity lesion in the right MO which indicating a banded infarction site (Fig. 1A). Fluid-attenuated inversion recovery images of brain MRI on T2 sequences on December 22, 2020 showed slight high-signal intensity in the infarcted site (Fig. 1B). What’s more, the magnetic resonance angiography on December 22, 2020 demonstrated that the left dominant vertebral artery which tortuously to the right with no abnormality was observed in the remaining cerebrovascular vessels (Fig. 1C). The patient was diagnosed with MO infarction, Avellis syndrome, Horner syndrome, dysphagia, and hemiparesthesia.

The patient was administrated aspirin to prevent the aggregation of platelet, rosuvastatin tablets to regulate lipids as well as to stabilize vascular plaque. She was injected with butylphthalide sodium chloride to improve nerve nutritional status. The patient again experienced electric shock-like pain radiating from the right throat to the ear root, temporal, and the orbital regions at 1 a.m. on December 22, 2020 which lasted for 30 minutes. Trigeminal neuralgia combined with GPN were taken into consideration and carbamazepine (0.1 g, bid) was prescribed. The patient reported no recurrence of electric shock-like pain in the right temporal as well as orbital region, while she still could feel occasional slight burning in the pharynx on December 25, 2020, and carbamazepine treatment was continued. The electric shock-like pain was completely gone after a period time of carbamazepine treatment. The numbness of the left side was significantly relieved, and the burning pain (with Visual Analogue Scale score of 6, self-reported as the sensation of the skin that soaked by hot pepper water) began to appear on the back of the left foot on January 3, 2021, then the abnormal sensation gradually extended to the whole left limbs, trunk, and face. The electromyography of extremities did not reveal abnormality and gabapentin was administrated. Dizziness, dysphagia, and Horner syndrome were significantly alleviated when she was discharged from the hospital, and secondary prevention of cerebral infarction was prescribed. All interventions during the hospitalization were well tolerated by the patient. The symptoms of dizziness, coughing while drinking and numbness of the left side were reported disappeared in the follow-up that 1 month after her discharge while the burning pain in the left body remained the same. The patient accepted several various treatments including pregabalin, antidepressants, and acupuncture in other hospitals regarding the abnormal burning pain but there was little effect as we were informed in the followed-up at 3rd, 6th, and 12th month after the discharge. There was no new lesion site found in her brain MRI and the electromyography of all limbs revealed no abnormality.

## 3. Discussion

The clinical manifestations of Avellis syndrome may vary depend on the lesion size and the involved nuclei as well as fibrous bundles. The nucleus ambiguus and the lateral spinothalamic tract are the most affected targets of Avellis syndrome in the case of MO infarction. However, in the case of MO infarction, the surrounding structures such as nucleus tractus spinalis nervi trigemini, nucleus of hypoglossal nerve, nucleus of accessory nerve, nucleus of the solitary tract, nucleus nervi vestibularis, trigeminal thalamic tract, and sympathetic fibers may be affected thus generate additional symptoms.^[7]^ Hoarseness, dysphagia, diminished sensation (light touch, pain, and temperature) of the contralateral face, trunk, and limbs are the most frequently reported clinical manifestations of Avellis syndrome. Hoarseness was reported in 9 cases of Avellis syndrome among the representative literatures^[3,5,7,8]^; dysphagia was reported in 6 cases; decreased sensation of the contralateral limbs or trunk was reported in 8 cases; diminished sensation of the contralateral face was reported in 4 cases; ipsilateral Horner syndrome and contralateral hemiplegia were mentioned in 4 cases and 1 case, respectively; ipsilateral peripheral facial paralysis which was due to the infarction of the pons was also mentioned in 1 case (see Table 1). In the current report, the patient presented with ipsilateral palatopharyngeal muscle palsy which could be explained by the affected nucleus ambiguous, glossopharyngeal nerve and the vagus; the occurrence of the diminished sensation of pain and temperature in the contralateral face may probably resulted from the damage to the ventral trigeminothalamic tract; the affected lateral spinothalamic tract may probably cause the sensation (pain and temperature) of the contralateral limbs and trunk to subside (Fig. 1D). In addition to the clinical manifestations, the cerebral MRI plays a significant role in the diagnosis of Avellis syndrome. The brain MRI images of this patient are consistent with what Takizawa, S, and Y Shinohara previously reported in a typical Avellis syndrome case which manifests as strip-like lesions that extending from the medial and lateral surfaces of the upper MO into the dorsimedial parenchyma which is usually demonstrated as decreased signal intensity on T1 sequences while increased signal intensity on T2 sequences.^[9]^ In general, the patient is affected by Avellis syndrome regarding the typical clinical manifestations as well as the cerebral MRI images.

**Table 1:** Avellis syndrome that caused by infarction of the MO.

Authors/year	Age/sex	Site that infarcted	Cause of symptoms	MRI results	Clinical manifestations
Kataoka et al^[7]^ (2001)	68/M	Left portion of the MO	Atherothrombotic disease in the territory of the distal vertebral artery	T2-weighted image 4d after onset of stroke showed a high-signal lesion in the left midlateral MO involving the right ambiguus nucleus, the lateral spinothalamic tract, and the ventral trigeminothalamic tract, including the ventral secondary ascending tract of the trigeminal nucleus. A small diagonal band shaped lesion was located in the upper medulla and extended from the midlateral surface to the deep parenchyma near the dorsal region of the rostral MO	Dysphagia and hoarseness; light touch and pain sensations were disturbed in right face, arm, trunk, and leg; left Horner syndrome
	42/M	Right portion of the MO	atherothrombosis of branches of the distal vertebral artery	A T2-weighted image 3d after onset of stroke revealed a high-signal lesion in the right midlateral MO, involving ambiguus nucleus and lateral spinothalamus tract. The wedge-shaped lesion extended from the midlateral surface to the deep parenchyma near the dorsal region of the MO	Dysphagia and hoarsenes; reduced sensations of light touch and pain were noted on trunk and left arm; right Horner syndrome
Habek et al^[3]^ (2007)	67/M	Right portion of the MO	brain stem arteritis because of neuroborreliosis	Transverse section at the level of MO showing high signal intensity in the right rostral portion	Swallowing difficulty and hoarseness; pain and temperature sensations were diminished on the left extremities; right Horner syndrome
Weigang et al^[8]^ (2018)	61/M	Right portion of the MO	Not mentioned	There was a strip lesion which was demonstrated as slight decreased-signal intensity on T1 sequences while high-signal intensity on T2 and DWI sequences on the left back of the MO. And there was a small dot lesion which was demonstrated as slight decreased-signal intensity on T1 sequences while high-signal intensity on T2 as well as DWI sequences	Dysphagia and hoarseness; decreased pain sensation in the right body; left peripheral facial paralysis
Kumral and Çetin^[5]^ (2021)	54/M	Right portion of the MO	Microscopic polyangiitis vasculitis	T2-weighted image showed a high-signal lesion in the right midlateral MO involving the right ambiguus nucleus, the lateral spinothalamic tract, lemniscus medialis, and the ventral ascending tract of the trigeminal nucleus (ventral trigeminothalamic tract	Hoarseness and dysphagia; reduced sensation of light touch and pain were noted on the left trunk, arm, and position; vibration sense of the upper limb was also decreased
	41/F	Right portion of the MO	Neuro-Behçet disease	T2-weighted image revealed a high-signal lesion in the right midlateral MO, involving ambiguus nucleus, lateral spinothalamus tract, and corticospinal tract	Hoarseness and dysphagia
	62/M	Left portion of the MO	Vertebral artery dissection	Magnetic resonance imaging showed a wedge-shaped lesion extending from the midlateral surface to the deep parenchyma near the dorsal region of the MO	Hypoesthesia and hypoalgesia; Pallhypesthesia were noted on the right face, arm, trunk, and legs; ocular lateropulsion; Gaze nystagmus; skew deviation
	69/M	Left portion of the MO	Small vessel disease	T2-weighted image demonstrated a left small diagonal band-shaped lesion located in the upper medulla and extended from the midlateral surface to the deep parenchyma involving the lateral spinothalamic tract, lemniscus medialis	Hoarseness and dysphagia; the sensation of light touch and pain was impaired in the right face, arm, and trunk; Horner syndrome; rotatory nystagmus; skew deviation
	57/F	Right portion of the MO	Neurobrucellosis small vessel vasculitis	A lesion in the right lateral part of the MO including nucleus ambiguous, medial lemniscus, hypoglossal tract, and corticospinal tract	Hoarseness; mild left brachiocrural hemiparesis; loss of sense of light touch, pain, and temperature

DWI = diffusion-weighted imaging, MO = medulla oblongata, MRI = magnetic resonance imaging.

In addition to the typical manifestations of Avellis syndrome described above, the patient also presented with electric shock pain in the right temporal, orbital, ear-root, and pharynx, as well as later burning pain in the left extremities, trunk, and face, which have not been reported before.

The patient felt sudden electric shock-like pain in the right temporal, orbital, root of ear and pharynx at 6th and 8th day since infarction which lasted dozens of minutes and then diminished automatically. According to the Chinese Expert Consensus on Diagnosis and Treatment of Trigeminal Neuralgia^[10]^ as well as the diagnostic criteria of GPN in International Classification of Headache Disorders (3rd edition).^[11]^ the patient may be probably tortured by TN and GPN. The simultaneous appearance of TN as well as GPN is still something of a rarity and the pressure on nerves generated by blood vessels is thought to be the most common cause.^[12]^ Warren et al^[13]^ reported a case of TN concurrent with GPN that secondary to lateral MO infarction, in which the initial symptom was the strong burning pain and the needling sensation from the left temple to the left ear, cheek, jaw, and pharynx. The high-resolution brain MRI demonstrated linear increased signal intensity on T2 sequences in the left spinotrigeminal nucleus as well as tract and left solitary nucleus which locates in the lateral part of the left medulla. A coronal 3D-CISS image demonstrated no compression of the trigeminal nerve at the pontine nerve root entry zone. The concurrence of TN as well as GPN was ascribed to the infarction, but the possible mechanism, however, was not discussed thoroughly. The peripheral branches of the trigeminal nerve are mainly composed of the ophthalmic nerve, maxillary nerve, and mandibular nerve, which distribute in the head and face and are responsible for the sensation of pain, temperature, and touch, among which the pain sensation is controlled by spinal trigeminal nucleus (STN). What’s more, the peripheral branches of the upper ganglion of glossopharyngeal nerve distribute in the outer ear skin as well as dura (responsible for the sensation of pain and temperature), whereas the central branches terminate in the STN. The STN is considered as the second-order neuron that is responsible for the transmission of oral and facial pain. Whether the damage to STN could result in the simultaneous occurrence of TN and GPN is of great value to be analyzed. It was reported by Trousseau^[14]^ in 1853 that epileptiform discharges were recorded in the midbrain during pain episodes in TN patient, and antiepileptic drugs (carbamazepine, phenytoin sodium, etc) were effective in controlling the pain; thus the idea that TN is a form of sensory epilepsy was developed. Jörg and Gerhard^[15]^ further pointed out that the pathological mechanism of TN was epileptic discharges in the STN which may be caused by the declined inhibiting effect. We propose that the concurrence of TN as well as GPN in this case was induced by the infarction of the MO which affects STN and cause epileptic discharge, which supports the concept that TN is a form of local epileptic discharge.

The patient was infarcted on the right side of the MO, presenting numbness of the left side when she was admitted into the hospital. The numbness gradually decreased with the treatment and disappeared eventually. However, the burning pain in the left foot and back appeared, which gradually extended to the left limbs, trunk, and face. The patient was diagnosed with central post-stroke pain (CPSP)^[16]^ considering the history, symptoms, signs, and imagings with other possible causes excluded. The pathogenesis of CPSP remains unclear. However, it is generally believed^[16–18]^ that the occurrence of CPSP is related to damage of several important anatomical structures, including the thalamus, spinothalamic tract, and anterior cingulate cortex. When structures such as thalamus, MO, and sensory cortex were involved in stroke, CPSP is more likely to occur. The pain of CPSP is restricted to the anatomical area with somatosensory abnormalities. The clinical manifestations are usually described as “burning pain,” “cooling sensation,” “needling sensation,” or “electric shock pain” in the face or trunk and limbs. Paresthesia may occur at the onset of stroke or months or years later especially at the time that the superficial sensory disorders of contralateral body begin to alleviate. The infarcted site in this case is MO. Studies^[16,19]^ have shown that lateral MO infarction is more likely to cause burning pain on the contralateral body, which may be caused by the stimulation to the lateral spinothalamic tract. The anatomic sequence of lateral spinothalamic tract from the lateral to the middle is sacral, lumbar, thoracic, and cervical, so patients with lateral MO infarction are prone to paresthesia of the lower limb at the onset and then upper limb paresthesia becomes obvious,^[20]^ which is consistent with this case. There lacks specific therapy in terms of CPSP. Drugs that could be used may include antidepressants, anticonvulsants, and opioids. Other methods such as mirror physiotherapy and noninvasive or invasive brain stimulation were reported.^[21]^ Gabapentin, pregabalin, and antidepressants showed little effect to the patient in this case. Long-term follow-up is still needed in case of deterioration of the burning pain and multi-disciplinary as well as multi-means combined therapy may be recommended.

In total, Avellis syndrome is a rarely reported clinical syndrome. The patient in this case presented with typical symptoms of Avellis syndrome such as palatopharyngeal paralysis, Horner syndrome, and diminished pain as well as temperature sensation in the contralateral face, trunk, and limbs, among which Horner syndrome should be differentiated from the dorsolateral MO syndrome, that is, Wallenberg syndrome because of the similar manifestations.^[2]^ The concurrence of ipsilateral TN as well as GPN caused by the damage to the STN and the CPSP of contralateral body caused by the affected lateral spinothalamic tract were for the first time reported in Avellis syndrome to our best knowledge.

## Acknowledgment

The authors wish to acknowledge the drafting support from Zhicong Jing.

## Author contributions

Conceptualization: Lihua Gu, Kaixuan Luo.

Data curation: Sijin He.

Investigation: Sijin He.

Methodology: Sijin He.

Project administration: Sijin He.

Resources: Qigang Chen.

Software: Zhicong Jing.

Supervision: Lihua Gu, Kaixuan Luo.

Writing – review & editing: Kaixuan Luo.
